# effect of 5-aminolaevulenic acid on postoperative lactate levels in patients undergoing surgery for malignant brain tumours

**DOI:** 10.1186/2197-425X-3-S1-A976

**Published:** 2015-10-01

**Authors:** G Baake, FE van Gelder, W Bult, MW Nijsten

**Affiliations:** Department of Critical Care, University Medical Center Groningen, Groningen, Netherlands; Hospital Pharmacy, University Medical Center Groningen, Groningen, Netherlands

## Introduction

5-aminolaevulinic acid (5-ALA) is a natural precursor of haemoglobin. Exogenously administered 5-ALA can lead to intracellular accumulation of fluorescent porphyrins in malignant tissues, such as glioblastoma. 5-ALA is increasingly used to improve tumour visualisation and enable more optimal resection of malignant gliomas. *In vitro*, 5-ALA can cause oxidative damage to rat liver mitochondria. *In vivo*, rats exposed to 5-ALA developed increased lactate levels; possibly because inhibition of oxidative metabolism [1]. Univariate data also suggested an effect of 5-ALA in neurosurgical patients [2]. Since lactate levels are widely used to monitor patients, we performed multivariate analysis on the impact of 5-ALA on lactate levels.

## Objectives

Asses the relation of preoperative 5-ALA on postoperative systemic lactate levels in patients undergoing surgery for malignant brain tumours.

## Methods

In an observational study in a cohort of neurosurgical patients who underwent resection of a suspected malignant glioma and were postoperatively admitted to our ICU, we compared lactate levels between patients who received 5-ALA preoperatively (5-ALA group) and those who did not (control group). The decision to use 5-ALA was at the discretion of the neurosurgeon and was based on the specific tumour characteristics on preoperative imaging. If fluorescent-guided resection was scheduled, the patient received 20 mg/kg of 5-ALA (Gliolan, Medac, Germany) orally 2 hours before the induction of anaesthesia. All patients received high-dose dexamethasone. Peri- and postoperative lactate and glucose levels were routinely obtained during the ICU stay in all included patients in this study using a point-of-care analyzer blood gas analyser.

## Results

From 2007 to 2014 we included 350 patients aged 56 ± 14, 60% males. 89 patiens (25%) received 5-ALA. These patients were older than controls (62 ± 8 vs. 53 ± 15;p < 0.001); duration of operation did not differ between the control and 5-ALA groups (NS). On day 0 the mean maximum lactate in the 5-ALA vs control groups was 2.83 ± 1.34 vs 2.47 ± 1.24 mmol/L (p = 0.02). On the first postoperative day the lactate levels were similar. Multivariate analysis showed that age (p = 0.02), duration of operation (p = 0.04) and glucose (p < 0.001), but not 5-ALA (p = 0.43) were related with lactate.

## Conclusions

5-ALA use was only univariately associated with increased postoperative lactate levels. MV-analysis, strongly points to a central role of hyperglycemia, as was recently also observed after cardiac surgery [3].
